# Dental Appointment Adherence Patterns in the College of Dentistry, King Saud Bin Abdulaziz University for Health Sciences: Insights From Electronic Dental Records

**DOI:** 10.7759/cureus.43677

**Published:** 2023-08-17

**Authors:** Hussam M Alqahtani, Yasmine N Alawaji

**Affiliations:** 1 Preventive Dental Science, College of Dentistry, King Saud Bin Abdulaziz University for Health Sciences, Riyadh, SAU; 2 Research and Development, King Abdullah International Medical Research Center, Riyadh, SAU; 3 Dental Hospital, Ministry of National Guard Health Affairs, Riyadh, SAU

**Keywords:** missed appointments, electronic health records, dental school, dental appointments, appointment adherence

## Abstract

Background

The purpose of this study was to determine the pattern of dental appointment adherence in the College of Dentistry (COD), King Saud Bin Abdulaziz University for Health Sciences (KSAU-HS).

Methodology

The electronic dental record SALUD (Two-Ten Health, Dublin, Ireland; *n* = 15,193) was used in this cross-sectional study. The primary outcome measure was adherence to dental appointments, categorized as attended, canceled by the patient, canceled by the school, or missed. Other variables of interest were demographic and appointment-related factors. Descriptive statistics were used to describe patterns of dental appointments. In addition, the proportion of check-in time for dental appointments among those who attended was calculated. For requested dental appointments among dental specialties, we calculated the percentage of booked, canceled, and rejected appointment requests for each specialty and compared the proportions across specialties.

Results

The proportion of attended dental appointments was 70.92% (10,775), with 9.14% (1,388) of appointments being missed and 16.70% (2,537) being canceled. Approximately 54% (5,765) of dental appointments were checked in on time. Approximately 77% (10,115) of dental appointment requests were scheduled. Pedodontics and orthodontics had the most scheduled appointments, while restorative dentistry had the most appointment requests.

Conclusions

The utilization of data from electronic dental records revealed a low rate of missed dental appointments. Identifying those who were late or skipped appointments was critical to determining the possible causes.

## Introduction

Adherence to scheduled appointments is a persistent problem in the healthcare system. Studies have shown that up to 30% of patients fail to keep their medical appointments [1-3]. In dentistry, the proportion of missed appointments was estimated at approximately 25% in a study conducted in the College of Dentistry at King Saud University [4]. Another study found that 58% of patients who had dental appointments in military hospitals in the Eastern Province missed their dental appointment [5]. However, relying on self-administered questionnaires to collect such data, as was the case in the previous studies, may introduce bias in the sample and responses. Therefore, the use of more reliable data would be of value in further understanding dental appointment adherence.

Clinical training of dental students is a crucial part of their education, and dental schools place a high value on clinical requirements to help students develop their skills. However, nonadherence to dental appointments not only has a significant negative impact on patients but also affects the training of dental students. To the best of our knowledge, no study has yet addressed this issue using electronic dental records to accurately estimate the frequency of dental appointment nonadherence in the College of Dentistry (COD) at King Saud Bin Abdulaziz University for Health Sciences (KSAU-HS). Therefore, shedding light on this issue is significant.

This study aimed to investigate dental appointment adherence patterns using electronic dental records at the COD, KSAU-HS. 

## Materials and methods

Data source and study population

This cross-sectional study was conducted in COD, KSAU-HS, during the first semester (August 2022 to December 2022) of the 2022-2023 academic year. The study included all patients who had dental appointments scheduled with students using the SALUD electronic dental record system. SALUD (Two-Ten Health, Dublin, Ireland) is a clinical management system that utilizes electronic dental records to improve patient care. It includes patient registration, appointment scheduling, treatment planning, and progress notes. Patients who presented as walk-ins or for an emergency visit were excluded from the study. This study was approved by the institutional review board of King Abdullah International Medical Research Center (# NRC22R/528/10).

Variables of interest

The primary variable of interest was adherence to a dental appointment, categorized as attended, canceled by the patient, canceled by the school, or missed. Demographic factors, such as patient age (grouped as <20, 20-30, 31-40, 41-50, 51-60, or >60 years) and sex (male or female), were also considered. The patient’s place of residence in Riyadh City was categorized according to the Riyadh Province region, which included north, west, center, south, and east, or classified as *other* for regions outside of Riyadh or those that were inaccurately labeled. The week number of the clinical session (1-17), the time of the clinical session (morning or afternoon), and the day of the week (Sunday, Monday, Tuesday, Wednesday, or Thursday) were recorded to describe the appointment status in detail. The check-in time of the dental appointment was also recorded and categorized as on time (within 30 minutes of the scheduled appointment) or late (after 30 minutes).

To assess the distribution of dental appointments across specialties, clinical specialties (endodontics, oral medicine, orthodontics, oral surgery, pedodontics, periodontics, prosthodontics, and restorative dentistry) and the outcome of the requested dental appointment (booked, canceled by students, or rejected by the receptionist) were included.

Statistical analysis

Descriptive statistics were used to describe patterns of dental appointments in COD, KSAU-HS. The percentage of patients with different appointment statuses (attended, canceled by the patient, canceled by the school, and missed) was compared with the different demographic and appointment-related categories. In addition, the check-in time for attended dental appointments was analyzed by calculating the proportion of on-time and late check-ins and examining the distribution of check-in time across different periods (morning and afternoon). Finally, to analyze the distribution of requested dental appointments among specialties, we calculated the percentage of booked, canceled, and rejected appointment requests for each specialty and compared the proportions across specialties.

## Results

The characteristics of dental appointments based on their appointment status are presented in Table 1. Of the 15,193 dental appointments, 1,389 (9.14%) were missed, while 10,779 (70.92%) were attended, 2,539 (16.70%) were canceled by the patients, and 493 (3.24%) received a cancellation from school.

**Table 1:** Characteristics of the study population. ^*^During the 11th week, only senior students were allowed to schedule patients. ^**^During these weeks, dental students had final exams and only who were late in their clinical requirements were allowed to schedule patients.

Variables	Appointment adherence				
	Attended	Canceled by patient	Canceled by school	Missed	Total
No. of dental appointment	10,775	2,537	493	1,388	15,193
Age (years), *n* (%)					
<20	3,153 (70.05)	764 (16.97)	181 (4.02)	403 (8.95)	4,501
20-30	2,702 (68.81)	717 (18.26)	136 (3.46)	372 (9.47)	3,927
30-40	2,062 (72.48)	432 (15.18)	62 (2.18)	289 (10.16)	2,845
40-50	1,632 (71.11)	426 (18.56)	57 (2.48)	180 (7.84)	2,295
50-60	774 (74.85)	117 (11.32)	42 (4.06)	101 (9.77)	1,034
>60	452 (76.48)	81 (13.71)	15 (2.54)	43 (7.28)	591
Sex, *n* (%)					
Male	5,840 (71.67)	1,235 (15.16)	259 (3.18)	815 (10.00)	8,149
Female	4,935 (70.06)	1,302 (18.48)	234 (3.32)	573 (8.13)	7,044
Patient’s location in relation to residence in Riyadh City, *n* (%)					
North	923 (68.37)	261 (19.33)	42 (3.11)	124 (9.19)	1,350
West	378 (66.06)	128 (22.74)	17 (2.89)	46 (8.30)	569
Center	164 (67.77)	41 (16.94)	7 (2.89)	30 (12.40)	242
South	485 (75.55)	74 (11.53)	18 (2.80)	65 (10.12)	642
East	4,663 (71.31)	1,092 (16.70)	202 (3.09)	582 (8.90)	6,539
Other	4,174 (71.16)	943 (16.08)	208 (3.55)	541 (9.22)	5,866
Week number, *n* (%)					
1	427 (59.72)	97 (13.57)	97 (13.57)	94 (13.15)	715
2	739 (75.95)	101 (10.38)	32 (3.29)	101 (10.38)	973
3	787 (74.53)	144 (13.64)	24 (2.27)	101 (9.56)	1,056
4	504 (76.83)	95 (14.48)	9 (1.37)	48 (7.32)	656
5	829 (74.75)	167 (15.06)	25 (2.25)	88 (7.94)	1,109
6	820 (73.41)	178 (15.94)	23 (2.06)	96 (8.59)	1,117
7	819 (74.86)	170 (15.54)	27 (2.47)	78 (7.13)	1,094
8	491 (69.94)	139 (19.80)	15 (2.14)	57 (8.12)	702
9	832 (70.09)	200 (16.85)	34 (2.86)	121 (10.19)	1,187
10	872 (68.77)	242 (19.09)	41 (3.23)	113 (8.91)	1,268
11^*^	331 (81.73)	4 (0.99)	42 (10.37)	28 (6.91)	405
12^**^	20 (90.91)	0 (0.00)	2 (9.09)	0 (0.00)	22
13^**^	2 (100.00)	0 (0.00)	0 (0.00)	0 (0.00)	2
14^**^	4 (80.00)	0 (0.00)	1 (20.00)	0 (0.00)	5
15	618 (69.13)	162 (18.12)	19 (2.13)	95 (10.63)	894
16	728 (63.41)	259 (22.56)	30 (2.61)	131 (11.41)	1,148
17	678 (67.53)	213 (21.22)	19 (1.89)	94 (9.36)	1,004
18	852 (69.38)	228 (18.57)	49 (3.99)	99 (8.06)	1,228
19	422 (71.40)	83 (14.04)	42 (7.11)	44 (7.45)	591
Time of the clinical session, *n* (%)					
AM	5,797 (69.65)	1,468 (17.64)	250 (3.00)	808 (9.71	8,323
PM	4,978 (72.93)	1,069 (15.56)	243 (3.54)	580 (8.44)	6,870
Day of the week, *n* (%)					
Sunday	2,073 (72.76)	432 (15.16)	81 (2.84)	263 (9.23)	2,849
Monday	2,512 (69.99)	585 (16.30)	158 (4.40)	334 (9.31)	3,589
Tuesday	1,661 (68.47)	450 (18.55)	86 (3.54)	229 (9.44)	2,426
Wednesday	2,109 (70.94)	522 (17.56)	74 (2.49)	268 (9.01)	2,973
Thursday	2,420 (72.11	548 (16.33)	94 (2.80)	294 (8.76)	3,356

Participants aged 30 to 40 years, males, those with a scheduled appointment in the first week, and those living in the central region of Riyadh Province had the highest percentage of missed dental appointments. In addition, elderly individuals and those living in the southern and eastern regions of Riyadh Province, as well as in regions outside of Riyadh or those with inaccurate labels, had the highest percentage of attended dental appointments. Conversely, individuals aged 20 to 30 years, residing in the western region of Riyadh Province, and having appointments scheduled in the first week displayed the lowest percentage of attended appointments. Furthermore, those residing in the eastern region of Riyadh Province, areas outside of Riyadh, or those with inaccurate labels, and who had appointments scheduled in the first week, exhibited the highest percentage of school-initiated cancellations.

The proportion of check-in times for attended dental appointments is presented in Table 2. Approximately 54% (3,157) of appointments were checked in on time, with most on-time check-ins occurring in the morning session. Meanwhile, most late check-ins were also observed in the morning session.

**Table 2:** Proportion of check-in timing of dental appointments.

	AM (*n *= 5,797), *n* (%)	PM (*n *= 4,978), *n* (%)	Total
Check-in time of the dental appointment			
On time	3,157 (54.76)	2,608 (45.24)	5,765
Late	2,640 (52.69)	2,370 (47.31)	5,010

Table 3 shows the distribution of dental appointment requests by dental specialty. Of the 13,154 requests for a dental appointment, 146 (1.11%) were rejected, 2,893 (22%) were canceled by dental students, and 10,115 (77%) were booked. Pedodontics and orthodontics had the highest percentage of booked appointments, while oral surgery, prosthodontics, and oral medicine had a higher percentage of appointments canceled by students. Restorative dentistry received the most appointment requests, followed by prosthodontics, while orthodontics had the lowest number of appointment requests.

**Table 3:** Distribution of dental appointments among/across clinical specialties.

Discipline	Booked, *n* (%)	Canceled by student, *n* (%)	Rejected, *n* (%)	Total
No. of requests	10,115 (76.90)	2,893 (21.99)	146 (1.11)	13,154
Endodontics	1,243 (76.97)	357 (22.11)	15 (0.93)	1,615
Oral medicine	1,196 (75.13)	376 (23.62)	20 (1.26)	1,592
Orthodontics	347 (80.32)	76 (17.59)	9 (2.08)	432
Oral surgery	799 (74.39)	267 (24.86)	8 (0.74)	1,074
Pedodontics	996 (80.58)	227 (18.37)	13 (1.05)	1,236
Periodontics	1,545 (77.17)	425 (21.23)	32 (1.60)	2,002
Prosthodontics	1,669 (75.69)	521 (23.63))	15 (0.68)	2,205
Restorative dentistry	2,320 (77.38)	644 (21.48)	34 (1.13)	2,998

## Discussion

This study found that 71% (10,775) of the scheduled dental appointments in COD, KSAU-HS, were attended, with 9% (1,388) being missed and 17% (2,537) being canceled by patients. The lower percentage of missed and canceled dental appointments in the current study compared with previous studies, which reported 24% missed and 40% canceled appointments in one study [4] and 58% missed and 54% canceled appointments in another study [5], can be explained by the use of electronic dental records and the large sample size, which together provide a better estimate of the proportion of dental appointment adherence.

Our results revealed that 17% (2,537) of scheduled dental appointments were canceled by patients, while 3% (439) were canceled by the school. This can be partially explained by late cancellations by patients, student absences due to illness, and errors in appointment scheduling. Therefore, identifying related factors may help reduce the frequency of cancellations and improve attendance rates for dental appointments. In addition, the check-in time of appointments was also explored. More than half of the appointments were attended on time and fell within the morning session. It is critical to the student's learning process to take full advantage of the time they have available to treat their patients. Therefore, it would be worth further investigation to target those who were late for their appointments.

This study found that adherence to dental appointments did not vary by clinical session time, day of the week, or gender. Our findings are consistent with earlier studies that revealed no gender differences [4,6,7]. However, older patients had a higher attendance rate than younger patients, which contradicts earlier research [4,6,7]. This result might be explained by the fact that older patients were underrepresented compared with patients younger than 60 years. In addition, patients who lived in the southern and eastern regions of Riyadh Province had a higher attendance rate, which may be due to the proximity of COD, KSAU-HS, in those areas. To reduce any potential bias from individuals residing in regions outside of Riyadh or those with inaccurate labels, they were categorized as *other*. Interestingly, such a category exhibited a significant attendance rate of 71% (4,174) based on a sample size of 5,866 individuals. We also looked at the distribution of dental appointments during the first 19 weeks of the current academic year. It should be mentioned that the COD KSAU-HS followed the three semesters recommended by the Ministry of Education. Therefore, there were very few appointments in the 12th to 14th weeks due to final exams and vacations, and senior students were only allowed to make appointments in the 11th week. In addition, it was observed that most missed appointments occurred in the first week. This could be attributed to the student’s failure to personally remind their patients of upcoming appointments, either because they had not yet come into contact with the patients or were unfamiliar with the scheduling system. It is worth noting that personal reminders have been proven to be an effective method for improving appointment attendance rates [8,9].

We also evaluated the distribution of dental appointments by specialty. Approximately 77% (10,115) of all requested appointments were scheduled. Restorative and prosthodontic specialties were most frequently requested, while pedodontics and orthodontics were most frequently approved. This could be because the comprehensive clinic covers all specialties except pedodontics and orthodontics, which could facilitate appointment scheduling since they only have a dedicated clinic during the week. Interestingly, 22% (2,893) of appointment requests were canceled by dental students, mainly in the specialties of oral surgery followed by prosthodontics and oral medicine. Due to a lack of data, the reasons for these cancellations could not be further investigated. However, it could be that lab work for prosthodontic cases is delayed or there is an emergency case that requires a change in the dental appointment. It should be noted that students may change the type of appointment to another dental specialty, as treatment plans may change during scheduled appointments. This may require further investigation to properly evaluate the distribution of specialty appointments.

The current study is the first known investigation to use electronic dental records to assess the extent of dental appointment adherence in Saudi Arabia. It included a sample size of 15,193 dental appointments in COD, KSAU-HS. The comprehensive data available to us allowed for a thorough description of the status of dental appointments. We examined patients' arrival times for their dental appointments, providing a resource for future studies to improve this aspect. We also analyzed the dental appointments requested and their distribution among clinical specialties to identify the specialties most in demand.

The significance of this study will help clinical affairs not only to identify the individuals who are more likely to miss or cancel their appointments but also to investigate the possible reasons for nonadherence. In addition, this study will help clinical affairs identify individuals who are more likely to keep an appointment to provide students with more clinical cases for the benefit of their training. Further, this study will emphasize the importance of screening visits in predicting attendance by explaining to patients exactly what procedures will be performed by students and how long they will take to meet patients' expectations.

Limitations of this study include the need for further investigation into the impacts of related variables. Future research should focus on assessing the distribution of dental appointment adherence among both new and regular patients. Moreover, the influence of the operator on appointment adherence was not explored in this study, warranting attention in subsequent research. Future studies should also consider stratifying appointment adherence to evaluate the potential impact of operator communication skills or the absence of reminders. Furthermore, it is important to acknowledge that this study was conducted exclusively at a single dental school in Saudi Arabia, which may limit its generalizability to the entire country's population.

## Conclusions

The attended dental appointments accounted for 71% (10,775), with 9% (1,388) being missed. Additionally, 54% (5,765) of dental appointments were checked in on time. Notably, pedodontics and orthodontics emerged with the highest number of scheduled appointments. This study highlights the patterns and factors associated with dental appointment adherence utilizing an electronic dental record. The findings of this study will help clinical affairs to identify patients who are less likely to attend their appointments to investigate the possible reasons for nonadherence.
